# Development and validation of a postgraduate anaesthesiology core curriculum based on Entrustable Professional Activities: a Delphi study

**DOI:** 10.3205/zma001345

**Published:** 2020-09-15

**Authors:** Parisa Moll-Khosrawi, Alexander Ganzhorn, Christian Zöllner, Leonie Schulte-Uentrop

**Affiliations:** 1 Universitätsklinik Hamburg Eppendorf, Klinik- und Poliklinik für Anästhesiologie, Hamburg, Germany

**Keywords:** core curriculum, anaesthetics, curriculum, postgraduate

## Abstract

**Background: **Postgraduate training curricula should not be based on time-spans or predefined numbers of performed procedures. One approach to link competencies to clinical tasks is the concept of Entrustable Professional Activities (EPA).

The goal of this study was the definition, ranking and validation of EPAs for anaesthesiology postgraduate training and the creation of an anaesthesiologic core curriculum.

**Methods: **Anaesthesiologists of different levels of training participated in the study (single-center, cross-sectional) . First, an expert group defined a preliminary list of EPAs. Then a first Delphi round (n= 47 participants) was applied to identify daily anaesthesiology tasks with the goal to define EPAs. From the first Delphi round a new set of EPAs was defined, using the template and mapping method. Through an alignment process, conducted by the expert group, the preliminary EPAs and the new set of EPAs from the first Delphi round were summarised into a new list of EPAs. This list was presented to the study participants in a second Delphi round (n=80 participants), with the goal to validate and rank each EPA and to define the year of entrustment. For this purpose, participants were asked in the second Delphi round if each EPA should be included into an anaesthesiology core curriculum and in which year of training entrustment should take place. Furthermore, they were asked to rank each EPA on a numeric scale, defining its importance. From this numeric scale, the content validity index (CVI) for each EPA was calculated.

Consensus of the results from the second Delphi round was calculated, using the one-way random effects model to calculate Intra-Class-Correlations (ICC). Percentages of agreement among the whole set of EPAs of this study and a previously published set of EPAs were computed.

**Results: **A core-curriculum comprising of 39 EPAs was developed. The EPAs were subdivided into superior/high and inferior/low scoring EPAs, reflecting their importance and were mapped to the year of entrustment. The results reached high consensus among the different participating anaesthesiologist groups (overall agreement was 0.96 for the CVI of each EPA and 0.83 for the year in which the EPAs should be entrusted). Agreement with the previously defined set of EPAs was 73.3%.

**Conclusion: **This study provides a further step in transforming postgraduate anaesthesiology training into a more contemporary approach. Other studies are necessary to complete and amend the presented core curriculum of EPA based postgraduate anaesthesiology training.

## Zusammenfassung

**Hintergrund: **Postgraduelle Weiterbildungscurricula sollten sich weder an Zeiten, noch an vorgegebenen Richtzahlen von durchgeführten Prozeduren orientieren. Ein vielversprechender Ansatz, um kompetenzbasierte Curricula in den klinischen Alltag zu integrieren, ist das Konzept der Entrustable Professional Activities (EPA).

**Methode:** Die Teilnehmer dieser monozentrischen Querschnittsstudie waren Anästhesiologen verschiedener Ausbildungsgrade. Im ersten Schritt wurde durch eine Expertengruppe eine vorläufige Liste von EPAs definiert. Im Anschluss wurde die erste Delphi-Runde (N=47 Teilnehmer) durchgeführt, um alltägliche anästhesiologische Tätigkeiten zu identifizieren. Aus den Ergebnissen der ersten Delphi-Runde wurde durch die Mapping-Methode und durch eine Template-Analyse eine weitere Liste an EPAs definiert. Diese Liste wurde den Studienteilnehmern in einer zweiten Delphi-Runde (N=80 Teilnehmer) mit dem Ziel präsentiert, jede EPA zu validieren, eine Reihung vorzunehmen und das Jahr, in welchem diese anvertraut werden sollte, zu definieren. Dazu wurden die Teilnehmer in der zweiten Runde zu jeder EPA befragt, ob diese Teil eines anästhesiologischen Kerncurriculums sein sollte und in welchem Jahr der Weiterbildung diese anvertraubar sein sollte. Desweiteren wurden die Teilnehmer gebeten, jede EPA auf einer numerischen Skala hinsichtlich ihrer Relevanz zu bewerten. Aus den Ergebnissen der numerischen Skala wurde der Content Validity Index (CVI) berechnet. Die Übereinstimmung der Ergebnisse in der zweiten Delphi-Runde wurde untersucht, indem das Paneldatenmodell angewendet wurde, um die Intraklassen-Korrelation (ICC) zu berechnen. Weiterhin wurde die prozentuale Übereinstimmung zwischen den EPAs dieser Studie und bereits publizierten EPAs berechnet.

**Ergebnisse:** Ein Kerncurriculum bestehend aus 39 EPAs wurde entwickelt. Die EPAs wurden entsprechend ihrer Relevanz in eine hoch und niedrig bewertete Gruppe unterteilt. Ferner wurde das Weiterbildungsjahr festgelegt, in welchem die EPAs anvertraut werden sollten. Die Ergebnisse zeigten eine hohe Übereinstimmung in den unterschiedlichen Gruppen von Anästhesisten (der CVI als Maß für die Zustimmung betrug 0.96 für die EPAs und 0.83 für das Ausbildungsjahr, in welchem die EPA anvertraut werden sollte). Die Übereinstimmung mit den publizierten EPAs betrug 73.3%.

**Schlussfolgerung: **Diese Studie ist ein weiterer Schritt, um die postgraduelle anästhesiologische Weiterbildung zeitgemäß zu strukturieren. Weitere Untersuchungen sind notwendig, um dieses EPA basierte anästhesiologische Kerncurriculum zu vervollständigen und anzupassen.

## 1. Background

Postgraduate medical curricula are traditionally constructed dependent on time, in which trainees spend a predefined period in training and do not have to prove their competencies [1]. Among experts, many votes emphasize competencies and request medical curricula to be not only the result of time-dependent knowledge acquisition [2], [3], [4]. Necessary competencies in the medical sector are defined by the CanMeds framework or the framework of the US Accreditation Council for Graduate Medical Education [5], [6]. These frameworks have been adopted worldwide for competency-based undergraduate and postgraduate education [1], [7].

In postgraduate specialist training, programmes are advised by the German Medical Association and defined by each regional medical board [8]. The German Medical Association amended the postgraduate anaesthesiology training guidelines, which were published in 2018 [9]. The regional boards have not released the final version of their programmes yet and the prerequisites for specialisation are still defined by training in predefined time spans and proof of conducted clinical procedures. 

The aim of the amendments was to focus more on outcome-based training [10] and therefore competencies were highlighted in the new training programmes. Yet, the broad implementation of the new competency-based training programmes might be difficult and is far from applicability in reality. This might be due to the fact, that competency-based medical education (CBME) is too theoretical and detached from daily practise and therefore competencies (descriptors of the quality of individual persons) are difficult to assess in the clinical work place [11], [12].

One solution approach to bridge the gap between competencies and clinical activities is the concept of Entrustable Professional Activities (EPAs). This concept operationalises CBME [13] by describing the work which is done in the clinical workplace and considers the required competencies. The need for EPA based curricula in undergraduate and postgraduate training has been expressed repeatedly [14], [15], [16].

An EPA is a subject-specific task or responsibility, which encompasses several competencies, knowledge and skills, that can be fully entrusted to a trainee [17]. An EPA must be measurable and observeable [13]. A fully described EPA consists of seven components [17]: Each EPA should have a precise “title”, reflecting the activity. The second component describes the “specifications and limitations“ of the activity, followed by the “relevant domains of competence“. Furhermore, the applied competency frameworks are mentioned. To clarify which knowledge, attitude and skills are expected of a trainee to carry out the EPA, the fourth component, “required experience, knowledge, skills, attitude and behavior“, is defined. As each EPA is a subject-specific task that can be fully entrusted to a trainee, prerequisites of entrustment need to be defined. This is included within the fifth component “assessment information sources to assess progress and ground a summative entrustment decision “. The fifth component also specifies which information is utilized by the supervisors to enable taking a summative entrustment decision. The sixth component describes at which stage of training entrustment for which level of supervision should be reached (1. Be present and observe, 2. Act with direct, pro-active supervision, 3. Act with indirect, re-active supervision, 4. Act with supervision not readily available, 5. Provide supervision to junior trainees). The last component of an EPA defines expiration dates of entrustment, if no preservation of competence for the EPA takes place.

An example for an EPA in anaesthesiology is “Providing anaesthetic care for extensive abdominal surgery”. The defined activities should include sufficient large units of professional practice, so that entrustment of the EPA marks a significant milestone for the trainee. Furthermore, due to continuous training, entrusted activities should be considered as units of professional practise which gain complexity with increasing levels of training. Therefore, to prevent the definition of many small EPAs, small EPAs can be nested within larger EPAs (nested EPAs), or practical procedures might be integrated into EPAs as “Observeable Practise Activities“ (OPAs) [17], [18]. An example for an OPA is “Insertion of a central venous line”. This practical procedure is necessary for various EPAs and must be entrusted as well [17], [19].

Although EPA-based training programmes have already been developed in other special branches (e.g. in psychiatry, orthopaedic surgery, gynaecology and paediatrics) [4], [20], [21], [22], training programmes in anaesthesiology have become more competency based, but have not been built on EPAs yet [23], [24], [25]. Only few departments have started to implement EPA based postgraduate training [https://sites.google.com/view/cbdwesternanes/list-of-epas], [https://www.anaesthesia.ie/epa/]. Jonker et al. describe EPA-based curricula in anaesthesiology in their *“Agenda for development and research”*, and claim that the first step should be to determine a consensus set of EPAs [19]. To our knowledge, only the published work of Wisman-Zwarter et al. defined a list of EPAs for postgraduate anaesthesiology training by consulting programme directors of anaesthesiology, and provided an example of how an existing curriculum can be transformed into an EPA-based curriculum [26]. Thus there is still a need to standardise curricula in anaesthesiology without just defining minimum numbers of tasks. 

In our single-center, cross-sectional, Delphi-based research study, we developed an EPA based core curriculum for postgraduate anaesthesiology training. Each EPA was mapped to the year of entrustment and the importance (ranking) of each EPA was defined. 

## 2. Methods

### 2.1. Study design and study participants 

This study was performed at the Department of Anaesthesiology at the University Medical Center of Hamburg-Eppendorf, Germany.

**Figure 1:**
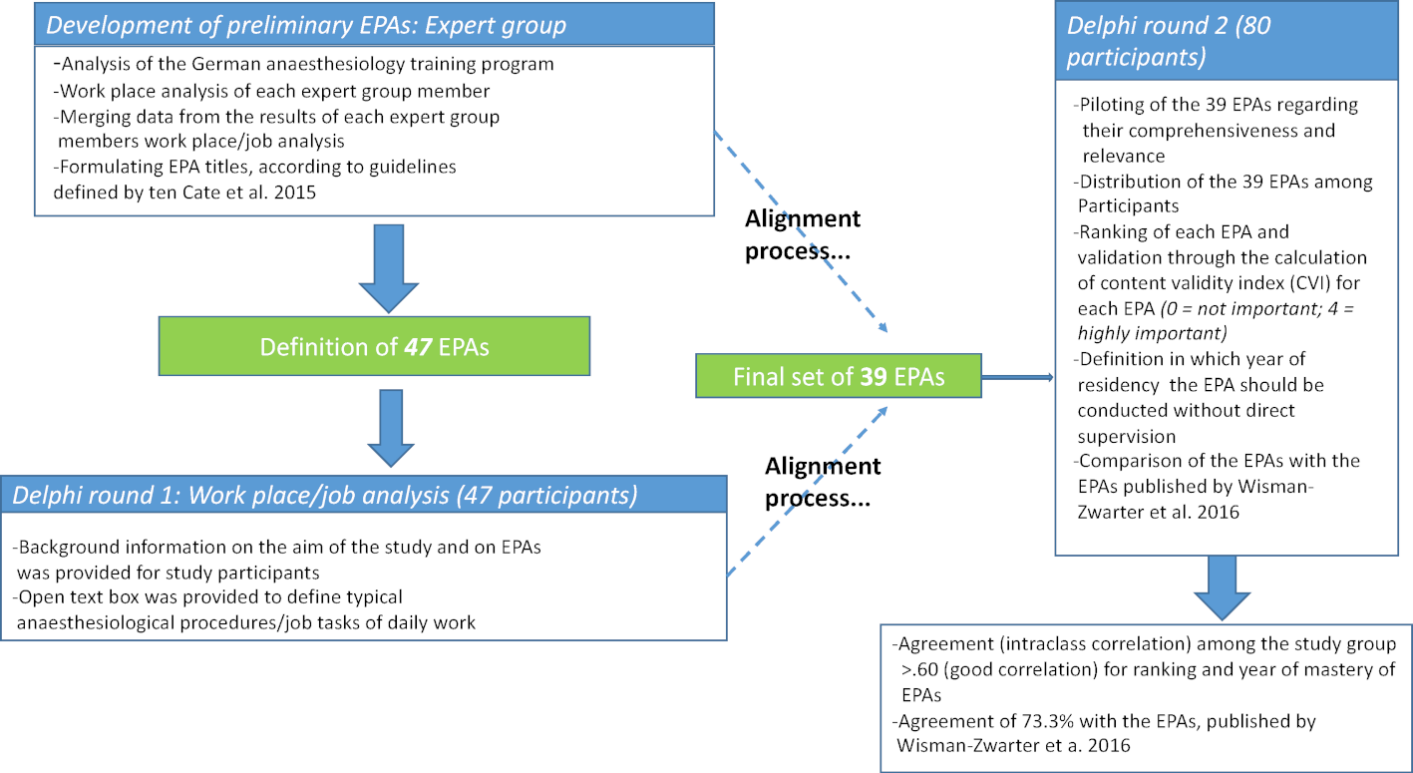
Study design and procedure

The design of our single-center, cross-sectional research study was a stepwise approach which included expert group analysis and the Delphi method to reach consensus among participants (see figure 1 (Fig. 1)). 

Expert group analysis is a qualitative research technique that has gained broad application in medical educational research [27], [28], [29], [30], [31], [32], [33]. The Delphi method is an iterative technique with the goal to reach group consensus and to collect expert opinion [34], [35]. 

The expert group consisted of three researchers and clinicians of different maturity, with profound knowledge in postgraduate training. Two of them are anaesthesiology specialists (consultant, attending, both female), one of them is second year resident (male). The mean age was 33.3 years.

In order to have both, the opinion of training experts of the department (consultants, supervising attendings), and of residents, all employees of the Department of Anaesthesiology (n=186) were eligible and invited to participate in the study. Data was analysed for the whole group of participants and subgroup analysis was conducted in order to detect differences between consultants, supervising attendings, regular attendings and residents of different years. The study size was defined by the number of participating employees. To prevent potential bias, members of the expert group did not participate in the Delphi study. 

An email explaining the study goal and providing background information on EPA was sent to all employees of the department (n=186) in April 2018. 

#### 2.2. Procedure

An overview of the study procedure is given in figure 1 (Fig. 1).

##### Development of preliminary EPAs (expert group)

First, the expert group analysed the current national anaesthesiology programme and outlined a preliminary list of EPAs. The EPAs were not fully described according to the 7 step-approach (AMEE Guide No.99, Ten Cate et al. [17]). As a first step to create an EPA based curriculum, the focus was to outline and define (precise title, step 1) daily anaesthesiology tasks, which have to be mastered during residency. The expert group made sure that the defined list of EPAs met all the necessary and realistic criteria for an EPA based curriculum and that the list of EPAs met all technical contents of the German anaesthesiology programme (provided by the German Medical Board) [8]. This programme defines minimum requirements of anaesthesiology procedures which have to be conducted during training, like 50 insertions of central venous lines or 25 fiberoptic intubations. All technical contents were considered, while the preliminary list of EPAs was developed.

Specialised pain medicine is not part of the core curriculum and therefore pain medicine was only included marginally within the preliminary list. Specific learning outcomes of intensive care medicine units (ICU) were excluded, because recent developments in the field of intensive care medicine focus on interdisciplinary patient care. Therefore, an interdisciplinary approach to define an ICU curriculum would be more effective and more representative for intensive care medicine in Germany. 

##### Delphi round 1: workplace/ job analysis

After the definition of the preliminary EPA list the first Delphi round was conducted. 

The study participants were provided with background information on the aim of the study and on EPAs based on the german publication of Breckwoldt et al. [36]. Then the participants listed possible EPAs by performing a brainstorming and analysing their daily work place. An open text box was provided to answer the question: *“Analyse and name daily anaesthesiological procedures (EPAs) that a resident has to face and master“*. The preliminary EPA list of the expert group was not disclosed to the study participants.

The data from this first Delphi round was analysed by the expert group and EPAs were extracted. Then the expert group conducted an adaptation process, comparing the defined EPAs of the first Delphi round with the preliminary list, resulting in a new list of EPAs. This new list was the basis for the second Delphi round. 

This final list was piloted by a group of 10 anaesthesiologists (7 residents, 3 attendings) to evaluate the clarity and comprehensiveness of the defined EPAs. After explaining the definition of the concept and common misunderstandings about EPAs [17], the piloting group was asked if the defined EPAs met the criteria of being an EPA (subject-specific task or responsibility). Further, they were askey if each EPA was comprehensible and relevant for anaesthesiology training. 

##### Delphi round 2:

In the second Delphi round the final list of EPAs was distributed among the study participants with the purpose of validating and ranking each EPA and defining the year of training in which entrustment (indirect supervision) should take place.

For this purpose, every department member received a survey and was asked three questions to each of the EPAs:

- Should this EPA be included into an anaesthesiology curriculum? *(Answers: “yes”, “no”)*
- Which rank would you give to this EPA concerning its importance for the anaesthesiology curriculum? (Ranks from 4= *“very important”*, to 1= *“not important“*).
- In which year of residency should this EPA be conducted without direct supervision? *(this question was explained with following citation: resident may act independently from supervisors only under postponed or backstage supervision* = step 6 of the guidelines provided by Ten Cate [17][) (choices between 1^st^ year, 2^nd^ year, 3^rd^ year, 4^th^ year, 5^th^ year, attending).

##### Comparison of the EPAs with the EPAs published by Wisman-Zwarter et al.

To provide a slight insight into whether a harmonisation of anaesthesiology training in Europe can be achieved, the final list of EPAs was compared to the list provided by the study of Wisman-Zwarter and collegues (Netherlands) [26].

#### 2.3. Statistical analysis

The qualitative data analysis (expert group analysis for the preliminary EPA list and the alignment process after Delphi round 1) was conducted with the template [37] and the mapping method [38]. (The template is presented in the supplement, see attachment 1 )

Statistical analysis was performed with SPSS (version 23.0, IBM Corp., Armonk, New York, USA). For all Delphi rounds, descriptive statistics were used for mean values, standard deviations and percentages. 

The content validity index (CVI) of each EPA was calculated for the ranking of the Delphi round 2, in which participants graded the EPAs by their importance (1= *“not important“* to 4= *“very important“*), reflecting the proportion of relevance [39]. A content validity index of 0.75 or higher is considered as “excellent“ [40]. 

Then, the EPAs were split into a “high ranking“ (CVI>0.75) and a “low ranking“ group (CVI<0.75) [41].

Mean scores and SDs were calculated for each question in Delphi round 2. Consensus among the groups was calculated, using the *one-way random effects model* to calculate Intra-Class-Correlations (ICC) [42]. The one-way random model was chosen, as the EPAs were rated by a random set of raters. Values of ICC below 0.40 are interpreted as poor correlation, between 0.40 and 0.59 as fair correlation, between 0.60 and 0.74 as good correlation and between 0.75 and 1.00 as excellent correlation [43].

For comparison of our findings with the data published by Wisman-Zwarter et al. [26], percentages of agreement among the whole set of EPAs were computed.

## 3. Results

### 3.1. Participants

Fourty-seven anaesthesiologists (25% response rate) participated in the Delphi round 1 and eighty (43% response rate) in the Delphi round 2. Table 1 (Tab. 1) shows the distribution of participation over the different training levels.

#### 3.2. Development of preliminary EPAs

The preliminary list of 47 EPAs, defined by the expert group, is shown in table 2 (Tab. 2).

#### 3.3. Delphi round 1: workplace/job analysis

214 answers from the workplace analyses were assigned to be EPAs and 182 responses were identified as OPAs, nested EPAs or other clinical activities by the expert group. From the 214 answers that met the criteria to be an EPA, a total of 30 EPAs was generated (see attachment 2 ). 

#### 3.4. Alignment process

After data analysis from Delphi round 1 was completed, the preliminary list of EPAs (n=47) was compared to the newly defined EPAs (n=30), resulting in some changes of the preliminary list. This adaptation and adjustment process, conducted by the expert group, resulted in a new list of 39 EPAs, integrating the new 30 EPAs. Some EPAs, which were not named at all or were part of another EPA, were excluded or summarised with other EPAs. Some of the EPAs were formulated very general, so they were splitted in several EPAs with a more finely granulation by the expert group.

A detailed description of the alignment process is provided in the supplement (see attachment 3 ).

##### Piloting of the final EPA list

The pilot group agreed on the importance of each EPA and stated that no relevant EPA was missing. It was confirmed that each EPA was comprehensive and met the required criteria.

#### 3.5. Delphi round 2

All participants agreed on the importance of each EPA and the 39 EPAs passed to the final set of EPAs (core curriculum).

The table in attachment 4 displays the content validity indices of each EPA, reflecting each EPAs` importance and dividing the EPAs into a superior/high score (23/39) and inferior/low score (16/39) group. The year of training in which participants declared the EPAs to be conducted without direct supervision is displayed.

**Figure 2:**
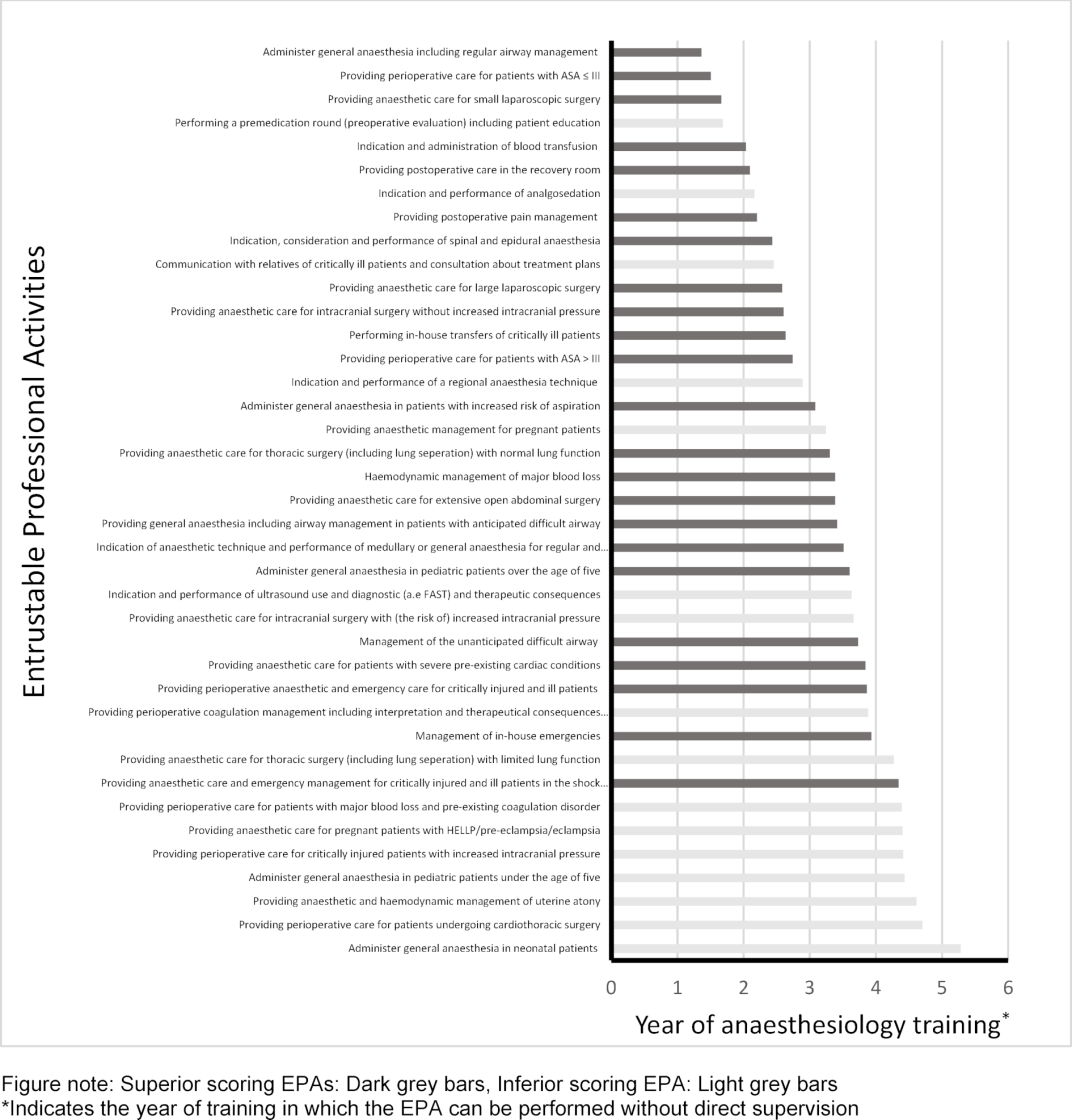
EPA core-curriculum proposal

The mapping of each EPA to a year of training, calculated by the mean value of participants´ rankings, resulted in an EPA core-curriculum proposal (see figure 2 (Fig. 2)).

The consensus among the groups (reflected by ICC) for the importance of each EPA and for the year of training in which each EPA should be entrusted, reached excellent levels of agreement. The overall agreement was 0.96 for the importance of each EPA and 0.83 for the year in which the EPAs should be conducted without direct supervision. The question at which stage which EPA should be conducted without direct supervision reached high consensus levels among the subgroups (>0.94). The consensus reflecting the importance of an EPA reached lower- but anyhow still good levels of agreement, comparing the group of consultants, supervising attendings and 1^st^ and 2^nd^ year residents (>0.63).

The levels of consensus calculated by ICC are shown in table 3 (Tab. 3).

#### 3.6. Comparison of the 39 EPA list with the EPA list of Wisman-Zwarter et al.

In a previous study, Wisman-Zwarter et al. [26] provided a list of 45 EPAs, which was generated through a national consensus procedure involving about 70% of all Dutch anaesthesiology programme directors. Our EPA list is not transferable one-by-one to the list of Wisman-Zwarter et al. [26]. Nevertheless, an agreement of 73.3% is noticeable. 26.6% of the list of Wisman-Zwarter et al. [26] is not included in our EPAs due to the fact that we have excluded EPAs concerning intensive care medicine and advanced pain management.

A detailed comparison of the two EPA lists is provided in the supplement (see attachment 5 ).

## 4. Discussion

In our study, we defined a list of 39 EPAs by an expert group and a consensus procedure (Delphi method) of 80 participating anaesthesiologists of different training levels. The 39 EPAs were ranked by their importance and the years of entrustment were defined. 

Regarding the AMEE guideline No.99 published by ten Cate et al. [17], which describes curriculum development bases on EPAs (seven steps), our study only completes two steps, namely defining EPA titles (step 1) and describing the year of entrustment (step 6). The goal of our study was to make a first step towards transforming the german anaesthesiology curriculum into an EPA based curriculum. The next steps should be to validate the curriculum nationwide and conduct some adaptations. Then, the remaining steps of defining an EPA curriculum should be completed. This would lead to a better validation and greater acceptence of the curriuculum and would faciliate its implementation.

A strength of our study is that the expert group consisted of consultant, attending and second year resident of the department – this diversity of the expert group was chosen to prevent potential bias. A consultant or a supervising attending might not identify problems that are perceived by residents. This fact differentiates our work from the study of Wisman-Zwarter et al., where only programme leaders participated which might have led to a partial and limited view of the subject [26].

As Wisman et al. pointed out, acceptance of an EPA-based core-curriculum should be achieved by a broad group of stakeholders – therefore, we chose anaesthesiologists in different stages of training, like attending clinicians (functioning as educators and supervisors) and trainees, who are mostly concerned by training curricula on a daily work basis. 

One might argue that the Delphi round 1 was unnecessary, because the responses only lead to 30 EPAs due to a possible unfamiliarity of the study participants with the concept of EPAs. Instead, the preliminary EPA-list of the expert group could have already been discussed in the first Delphi round. To bypass the relative unfamiliarity of the participants with the concept of EPAs, they received a detailed description prior to the study and shortly prior to Delphi round 1 and verbal explanations were provided by the expert group. 

Taking into account that the first Delphi round of our study functioned as work-place analysis of different anaesthesiologists in different stages of training, the objection of redundancy of Delphi round 1 can be overruled. For further studies it should be considered, that the unfamiliarity of study participants with the concept of EPAs leads to time consuming data analysis and the discussion of preliminary lists might be more efficient. 

The analysis of the first Delphi round resulted in changes of the preliminary EPA list. An adaptation process was conducted, in which some EPAs were merged and some were subdivided into more finely granulated EPAs, resulting in a total of 39 EPAs. These adaptations were possible, without restricting the German core-curriculum of the German Medical Association (excluding learning objectives of ICU). The refining of the expert groups´ preliminary list allowed scrutinising possible core EPAs from different perspectives and therefore resulted in a more realistic depiction of every day EPAs. For example, in one adaptation step, the focus was taken away from the perioperative risk and towards the patient itself. The rationale for this merging was that for an ASA IV patient it is indeed relevant if he undergoes a whipple resection or just a cholecystectomy. Nevertheless, the trainee should be entrusted with giving care to the particular patient itself. 

Surgical steps and interventions, which can result in anaesthesiological interference, must also be entrusted to the trainee without focusing on the ASA classification. 

Participants´ agreement regarding the importance (CVI) [40], [41] of each EPA and the year of training in which each EPA should be entrusted to the trainee (level IV) achieved almost excellent levels of correlation (ICC) [43]. Only comparison of the group of supervising consultants/ attendings and first and second year residents achieved “good” (not excellent) correlation regarding the importance (CVI) of each EPA. This might be due to different perspectives on the EPAs, based on the different experience levels. The EPAs with the highest discrepancies were* “Performing in-house transfers of critically ill patients”*, *“Providing postoperative care in the recovery room”* and *“Performing a premedication round (preoperative evaluation) including patient education”*. One explanation might be the Dunning-Kruger effect, a cognitive bias in which the junior residents (low ability at an EPA) overestimate their ability [44]. The junior residents might not realise the importance and possible adverse events of those activities (EPAs). The results of the second Delphi round, reflecting the year in which each EPA should be entrusted, support this theory: nearly all years of training in which entrustment should take place is stated at a lower level of training by the residents, than by the attendings and consultants. 

One limitation of our study is the single-center design. This might cause difficulties in using our results for support of a curriculum reform by other faculties [45]. Furthermore, the expert group members and the study participants work at a university medical center which is a maximum care hospital that could have biased some results. For example the EPA “Providing perioperative care for patients undergoing cardiothoracic surgery“, does not reflect common anaesthesiology skills, necessary to achieve specialist qualification, as many regional hospitals do not have a cardiothoracic surgery department. Therefore, a nationwide validation of the EPA curriculum is even more important. Many faculties could contribute by conducting a similar study to a broader, maybe even international consensus. 

## 5. Conclusions

Our study presents a proposed list of EPAs that describes postgraduate training in anaesthesiology. The list includes each EPAs` importance (CVI) and the anaesthesiology training year in which level IV (entrustment) should be achieved by the trainee. 

Further validation of the EPAs should take place by a larger number of stakeholders, including programme directors, determining a broader consensus of the EPAs. Then, each EPA should be completed based on the AMEE guideline no.99, which describes a seven-step approach for curriculum development for workplaces based on EPAs [17]. 

Subsequent studies should investigate, whether anaesthesiology training in Europe can be homogenised. For a start, we found a 73.3% accordance with the results of the Dutch colleagues [26]. Our study provides a reproducible approach of how EPA based curricula can be developed. 

With this study, a further step is made to transform current postgraduate anaesthesiology training to a more contemporary approach to prevent that the concept of EPAs becomes another buzzword in medicine [46].

## Abbreviations

- EPA: Entrustable Professional Activity
- OPA: Observable Practise Activity
- SD: Standard deviation
- MV: Mean value
- CVI: Content validity index
- ICC: Intraclass correlation
- ASA: American Society of Anesthesiology
- YR: Year of entrustment

## Declarations

### Ethics approval and consent to participate

The local ethics committee of Hamburg (Ethikkommission der Ärztekammer Hamburg, Hamburg, Germany) received a detailed project description and approved the project (no necessity of appraisal). Participants declared their consent to participate by participating (this information was provided alongside the email which explained the study goal). This consent of participation by participation was included within the project description for the ethics committee and was approved. 

#### Availability of data and materials

The datasets used and/or analysed during the current study are available from the corresponding author on reasonable request. The supplement contains nearly all data.

## Authors' contributions

All listed authors have read and approved the manuscript.

*PM-K* made substantial contributions to conception and design, as well as acquisition, analysis and interpretation of data. She has been involved in drafting and revising the manuscript and given final approval of the version to be published. She has been involved in drafting the manuscript and given final approval of the version to be published. She agreed to be accountable for all aspects of the work in ensuring that questions related to the accuracy or integrity of any part of the work are appropriately investigated and resolved.

AG made substantial contributions to conception and design, as well as acquisition, analysis and interpretation of data. He has been involved in drafting and revising the manuscript and given final approval of the version to be published. He agreed to be accountable for all aspects of the work in ensuring that questions related to the accuracy or integrity of any part of the work are appropriately investigated and resolved.

*CZ* made substantial contributions to conception and design, analysis and interpretation of data. He has been involved in drafting the manuscript and revising it critically for important intellectual content. He has given final approval of the version to be published. He agreed to be accountable for all aspects of the work in ensuring that questions related to the accuracy or integrity of any part of the work are appropriately investigated and resolved.

*LS-U* made substantial contributions to acquisition of data. She has been involved in revising the manuscript critically for important intellectual content and has given final approval of the version to be published. She agreed to be accountable for all aspects of the work in ensuring that questions related to the accuracy or integrity of any part of the work are appropriately investigated and resolved.

The authors Parisa Moll-Khosrawi and Alexander Ganzhorn contributed equally. 

## Acknowledgements

We would like to thank the anaesthesiologists of the Department of Anaesthesiology, University Medical Center Hamburg Eppendorf for their participation.

## Competing interests

The authors declare that they have no competing interests. 

## Supplementary Material

Template of the qualitative data analysis of Delphi round 1

The 30 EPAs generated from the first Delphi round

Alignment process of the preliminary EPAs

Content validity indices of all EPAs and the Year of indirect supervision

Comparison of our 39 EPA list with the EPA list of Wisman-Zwarter et al

## Figures and Tables

**Table 1:**
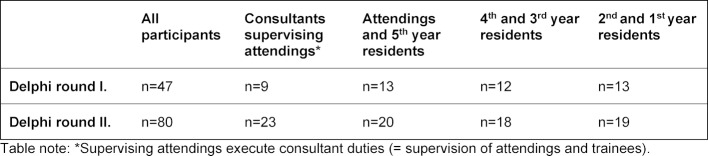
Number and anaesthesiology training of all participants in each Delphi round

**Table 2:**
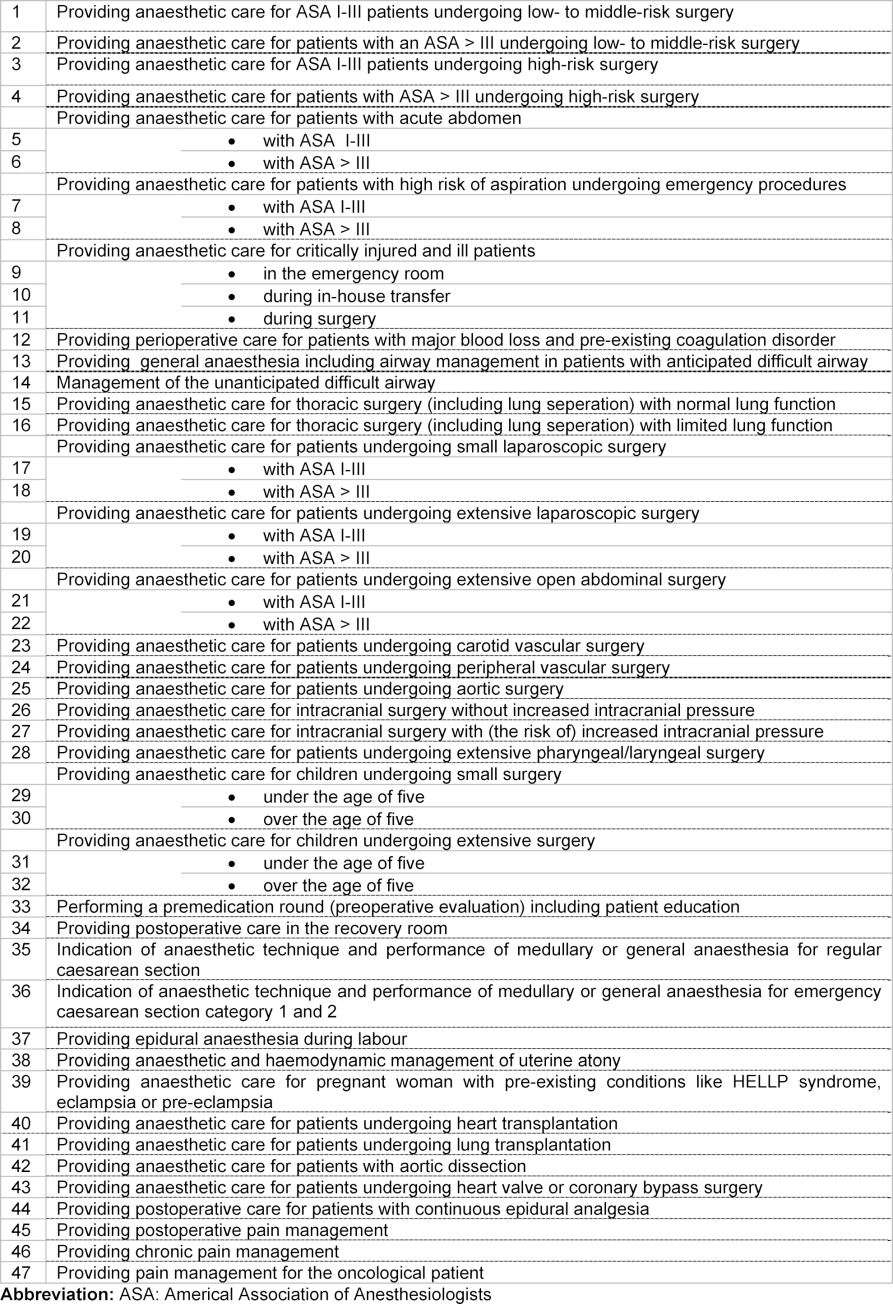
Preliminary list of EPAs

**Table 3:**
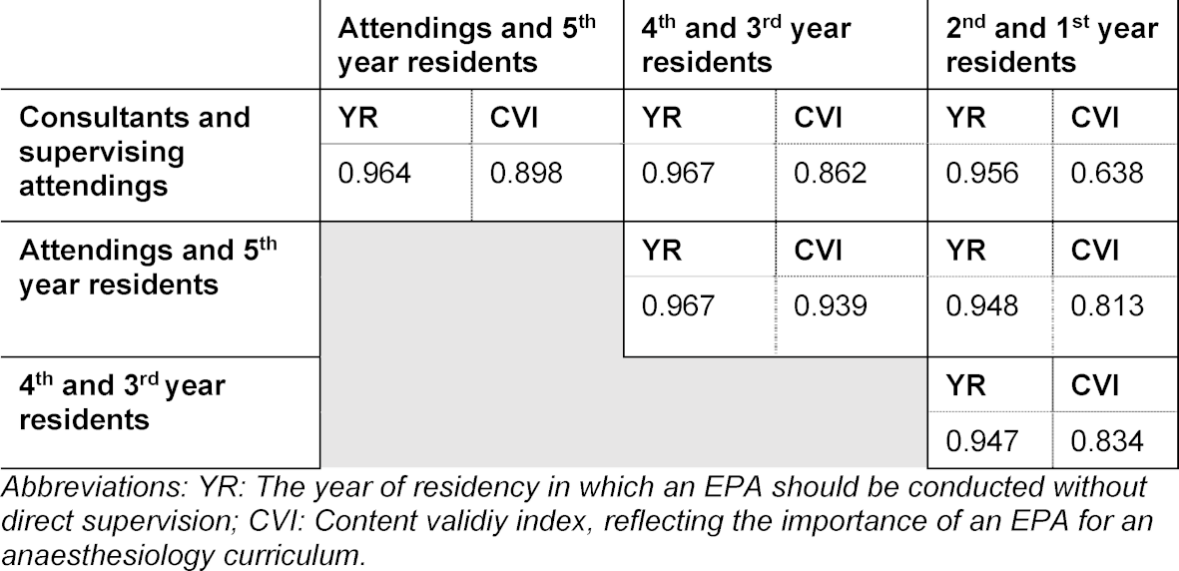
Consensus among the group of participants
