# Supplementary material for: Development and validation of a postgraduate anaesthesiology core curriculum based on Entrustable Professional Activities: a Delphi study
Source: GMS J Med Educ. 2020 Sep 15;37(5):Doc52. doi: 10.3205/zma001345 (PMC7499458; doi:10.3205/zma001345)
Supplement: Template of the qualitative data analysis of Delphi round 1 [file JME-37-52-s-001.pdf]

Attachment_1: Template of the qualitative data analysis of Delphi round 1

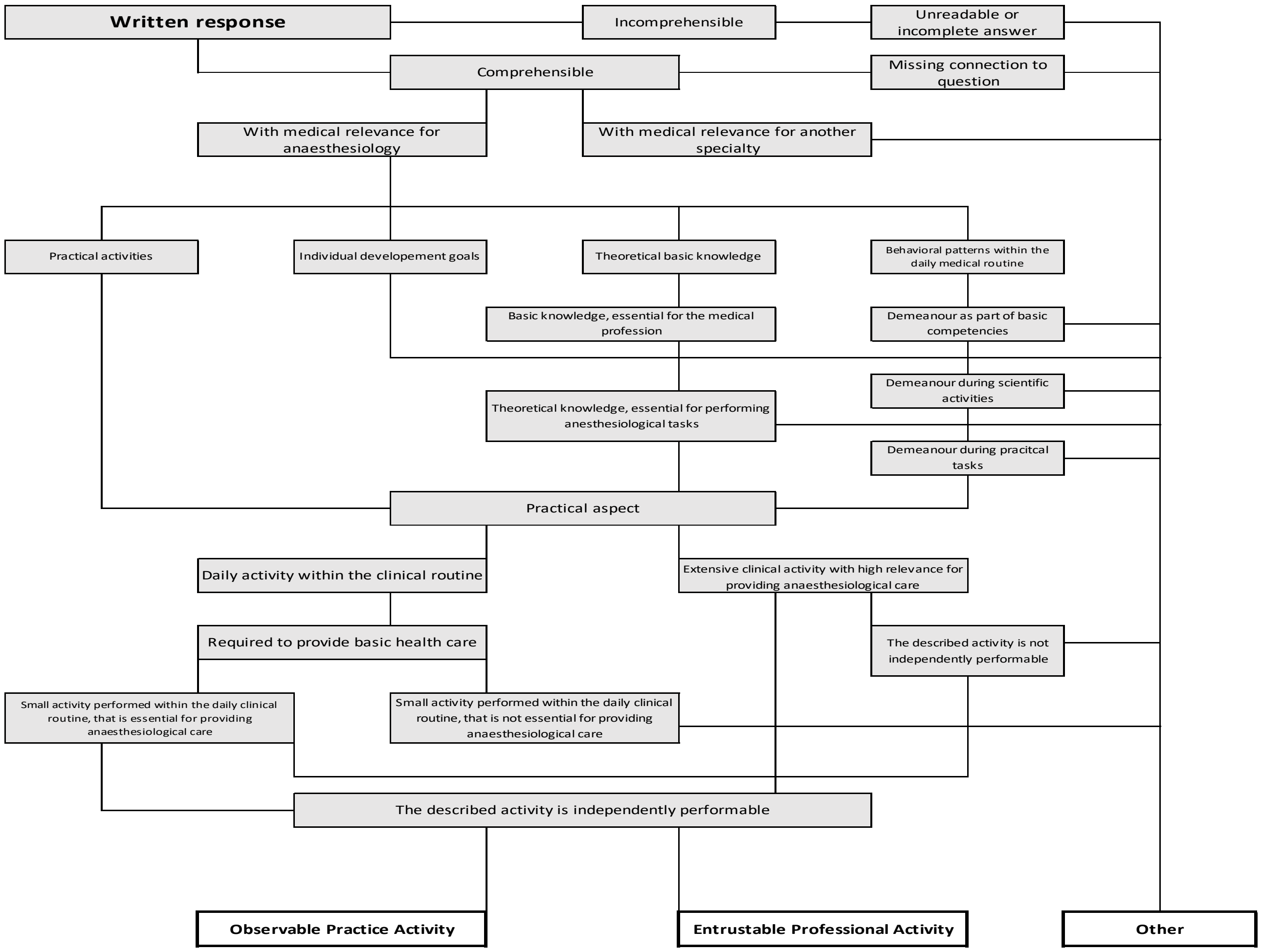

Attachment 1 to: Moll-Khosrawi P, Ganzhorn A, Zöllner C, Schulte-Uentrop L. *Development and validation of a postgraduate anaesthesiology core curriculum based on Entrustable Professional Activities: a Delphi study.* GMS J Med Educ. 2020;37(5):Doc52. DOI: 10.3205/zma001345

1.0 Quality of response

- 1.1 Comprehensible
  - 1.1.1 Missing connection to question
- 1.2 Incomprehensible
  - 1.2.1 Unreadable or incomplete answer

2.0 Relevance within the clinical context

- 2.1 With medical relevance for anaesthesiology
- 2.2 With medical relevance for another specialty

3.0 Categorization of the responses

- 3.1 Behavioral patterns within the daily medical routine
  - 3.1.1 Demeanour during pracitcal tasks
  - 3.1.2 Demeanour as part of basic competencies
  - 3.1.3 Demeanour during scientific activities
- 3.2 Individual developement goals
- 3.3 Theoretical basic knowledge
  - 3.3.1 Basic knowledge, essential for the medical profession
  - 3.3.2 Theoretical knowledge, essential for performing anesthesiological tasks
- 3.4 Practical activities

4.0 Practical aspect

- 4.1 Daily activity within the clinical routine
  - 4.1.1 Required to provide basic health care
    - 4.1.1.1 Small activity performed within the daily clinical routine, that is essential for providing anaesthesiological care
    - 4.1.1.2 Small activity performed within the daily clinical routine, that is not essential for providing anaesthesiological care
- 4.2 Extensive clinical activity with high relevance for providing anaesthesiological care

5.0 Independently performable activity

- 5.1 The described activity is independently performable
- 5.2 The described activity is not independently performable

Attachment 1 to: Moll-Khosrawi P, Ganzhorn A, Zöllner C, Schulte-Uentrop L. *Development and validation of a postgraduate anaesthesiology core curriculum based on Entrustable Professional Activities: a Delphi study.* GMS J Med Educ. 2020;37(5):Doc52. DOI: 10.3205/zma001345
