# Supplementary material for: Development and validation of a postgraduate anaesthesiology core curriculum based on Entrustable Professional Activities: a Delphi study
Source: GMS J Med Educ. 2020 Sep 15;37(5):Doc52. doi: 10.3205/zma001345 (PMC7499458; doi:10.3205/zma001345)
Supplement: The 30 EPAs generated from the first Delphi round [file JME-37-52-s-002.pdf]

Attachment 2: *The 30 EPAs generated from the first Delphi round*

**Entrustable Professional Activity**	**Namings**
Administer general anaesthesia including regular airway management	19.6%
Providing in-house emergency management	15.4%
Providing general anaesthesia including airway management in patients with anticipated difficult airway	14.0%
Management of the unanticipated difficult airway	14.0%
Performing a premedication round including patient education	10.3%
Indication and administration of blood transfusion	8.4%
Providing perioperative coagulation management	7.5%
Administer general anaesthesia in paediatric patients	6.1%
Providing postoperative pain management	6.1%
Performing haemodynamic monitoring with therapeutical consequences	6.1%
Indication and performance of an analgosedation	5.1%
Providing perioperative anaesthetic and emergency care for critically injured and ill patients	5.1%
Providing fluid management including differentiated infusion therapy	4.7%
Indication and performance of ultrasound use and diagnostic (a.e FAST) and therapeutic consequences	4.2%
Indication and performance of regional anaesthesia techniques	3.7%
Providing general and medullary anaesthesia in the labor room	3.7%

Attachment 2 to: Moll-Khosrawi P, Ganzhorn A, Zöllner C, Schulte-Uentrop L. *Development and validation of a postgraduate anaesthesiology core curriculum based on Entrustable Professional Activities: a Delphi study.* GMS J Med Educ. 2020;37(5):Doc52. DOI: 10.3205/zma001345

Providing anaesthetic management for pregnant patients	2.3%
Providing postoperative care in the recovery room	1.9%
Providing perioperative care for patients with ASA > III	1.9%
Providing perioperative care for patients with major blood loss	1.9%
Performing communication with relatives of critically ill patients and consultation about treatment plans	1.4%
Providing anaesthetic care for thoracic surgery (including lung seperation)	1.4%
Providing anaesthetic care for elderly patients (geriatric)	1.4%
Providing perioperative care for patients with ASA I-III	1.4%
Providing anaesthetic care for intracranial surgery without increased intracranial pressure	0.9%
Providing anaesthetic care for patients undergoing low- to middle-risk surgery	0.9%
Providing anaesthetic care for patients with crucial pre-existing cardiac conditions	0.9%
Performing in-house transfers of critically ill patients	0.5%
Providing anaesthetic care for patients undergoing high-risk surgery	0.5%
Providing perioperative care for critically injured patients with increased intracranial pressure	0.5%
