# Supplementary material for: Development and validation of a postgraduate anaesthesiology core curriculum based on Entrustable Professional Activities: a Delphi study
Source: GMS J Med Educ. 2020 Sep 15;37(5):Doc52. doi: 10.3205/zma001345 (PMC7499458; doi:10.3205/zma001345)
Supplement: Alignment process of the preliminary EPAs [file JME-37-52-s-003.pdf]

Attachment 3: Alignment process of the preliminary EPAs

The predefined EPAs *“Providing anaesthetic care for ASA I-III patients undergoing low to middle risk surgery”, “Providing anaesthetic care for patients with ASA > III undergoing low to middle risk surgery”, “Providing anaesthetic care for ASA I-III patients undergoing high risk surgery”, “Providing anaesthetic care for patients with ASA > III undergoing high risk surgery”*, were merged to “*Providing perioperative care for patients with ASA score > III” and* “*Providing perioperative care for patients with ASA score I-III”.*
The ASA state of the patient was deleted in the EPAs of *“Providing anaesthetic care for patients undergoing small laparoscopic surgery with ASA I-III / with ASA > III”* and *“Providing anaesthetic care for patients undergoing extensive laparoscopic surgery with ASA I-III / with ASA > III”* and within the EPA “*Provide anaesthetic care for extensive open abdominal surgery”.*

The preliminary EPAs “*Providing anaesthetic care for patients with acute abdomen with ASA I-III / with ASA > III”* and “*Providing anaesthetic care for patient with high risk of aspiration undergoing emergency procedures with ASA I-III / with ASA > III”,* were merged to *“Administer general anaesthesia in patients with increased risk of aspiration”*, due the results of delphi round I., in which providing care for patients with increased risk of aspiration was mentioned far more frequently than providing care for patients with acute abdomen.
Here, the focus was taken away from the patients´ condition that requires a rapid sequence induction (RSI), to the procedure of performing an RSI.

Due to the high frequency of statements in Delphi round I., the predefined EPA *“Management of patients with severe blood loss and pre-existing coagulation disorder”* resulted in two new EPAs *“Providing perioperative care for patients with major blood loss and pre-existing coagulation disorder”* and *“Providing perioperative coagulation management including interpretation and therapeutical consequences of thrombelastometry”.*

In the pre-defined EPA list orphan anaesthesia was subdivided into the pediatric patients age and the kind of surgery which is performed. For the new list, these EPAs were merged and only subclassified into the pediatric patients age.

"Indication of anaesthetic technique and performance of medullary or general anaesthesia for regular caesarean section", "*Indication of anaesthetic technique and performance of medullary or general anaesthesia for emergency caesarean section category 1 and 2*" and "*Providing epidural anaesthesia during labour*" were merged to *"Indication of anaesthetic technique and performance of medullary or general anaesthesia for regular and emergency caesarean section"*, as this new EPA includes all aspects of the aforementioned.

The preliminary EPAs of the field of cardiac anaesthesia were very finely granulated by the expert group, which was merged to just one EPA. The same procedure was conducted for pain medicine. The expert group agreed on this, as cardiac anaesthesia is not required in the core-curriculum, defined by the German Medical Board. Further, pain medicine has to be conducted during specialist training on basic level and it is also not part of the core-curriculum. The EPAs *"Providing anaesthetic care for patients undergoing carotid vascular surgery ", "Providing anaesthetic care for patients undergoing peripheral vascular surgery"* and *"Providing anaesthetic care for patients undergoing aortic surgery"* were deleted from the list, as they were not mentioned frequently enough in Delphi round I.

"*Providing anaesthetic care for patients undergoing oncological pharyngeal/laryngeal surgery*" was deleted, as the necessary skills (mainly difficult airway management) are represented in the EPA "*Providing general anaesthesia including airway management in patients with anticipated difficult airway*".
