# Supplementary material for: Development and validation of a postgraduate anaesthesiology core curriculum based on Entrustable Professional Activities: a Delphi study
Source: GMS J Med Educ. 2020 Sep 15;37(5):Doc52. doi: 10.3205/zma001345 (PMC7499458; doi:10.3205/zma001345)
Supplement: Content validity indices of all EPAs and the Year of indirect supervision [file JME-37-52-s-004.pdf]

Attachment_4: *Content validity indices of all EPAs and the Year of indirect supervision*

	**General**			**Consultants and supervising attendings**			**Attendings and 5th year residents**			**4th and 3rd year residents**			**1st and 2nd year residents**		
**Entrustable Professional Activity**	***CVI***	***MV ± SD***	***YR ± SD***	***CVI***	***MV ± SD***	***YR ± SD***	***CVI***	***MV ± SD***	***YR ± SD***	***CVI***	***MV ± SD***	***YR ± SD***	***CVI***	***MV ± SD***	***YR ± SD***
**Administer general anaesthesia in patients with increased risk of aspiration**	**0.96**	3.68 ±0.54	3.08 ±1.59	**0.96**	3.70 ±0.55	3.00 ±1.44	**0.95**	3.65 ±0.57	3.26 ±1.68	**1.00**	3.72 ±0.45	3.33 ±1.37	**0.95**	3.63 ±0.58	2.74 ±1.77
**Haemodynamic management of major blood loss**	**0.94**	3.60 ±0.64	3.38 ±1.15	**0.87**	3.39 ±0.82	3.64 ±1.07	**1.00**	3.75 ±0.43	3.35 ±1.19	**0.94**	3.61 ±0.59	3.56 ±0.96	**0.95**	3.68 ±0.57	2.95 ±1.23
**Administer general anaesthesia including airway management in patients with anticipated difficult airway**	**0.94**	3.56 ±0.65	3.41 ±1.36	**0.87**	3.35 ±0.81	3.43 ±1.06	**1.00**	3.65 ±0.48	3.50 ±1.32	**0.89**	3.56 ±0.68	3.67 ±1.29	**1.00**	3.74 ±0.44	3.05 ±1.67
**Providing anaesthetic care for extensive open abdominal surgery**	**0.94**	3.49 ±0.61	3.38 ±0.90	**0.96**	3.57 ±0.58	3.35 ±0.76	**0.95**	3.40 ±0.58	3.40 ±0.97	**0.94**	3.56 ±0.60	3.44 ±0.90	**0.89**	3.42 ±0.67	3.32 ±0.98
**Administer general anaesthesia including regular airway management**	**0.93**	3.71 ±0.60	1.36 ±0.62	**0.96**	3.83 ±0.48	1.43 ±0.58	**0.95**	3.80 ±0.51	1.35 ±0.57	**0.89**	3.56 ±0.68	1.44 ±0.76	**0.89**	3.63 ±0.67	1.21 ±0.52
**Indication and administration of blood transfusion**	**0.91**	3.48 ±0.81	2.03 ±0.91	**0.91**	3.48 ±0.93	2.04 ±0.86	**0.95**	3.40 ±0.73	2.10 ±0.77	**0.94**	3.61 ±0.59	2.22 ±0.71	**0.84**	3.42 ±0.88	1.74 ±1.16
**Management of the unanticipated difficult airway**	**0.89**	3.60 ±0.78	3.73 ±1.64	**0.87**	3.52 ±1.02	3.78 ±1.72	**0.90**	3.60 ±0.66	4.05 ±1.40	**0.94**	3.61 ±0.59	3.78 ±1.47	**0.84**	3.68 ±0.73	3.26 ±1.80
**Providing perioperative care for patients with ASA > III**	**0.89**	3.41 ±0.79	2.74 ±1.05	**0.83**	3.13 ±0.90	3.09 ±1.06	**0.90**	3.45 ±0.80	2.50 ±0.74	**0.94**	3.56 ±0.60	2.83 ±0.90	**0.89**	3.58 ±0.67	2.47 ±1.27
**Indication of anaesthetic technique and performance of medullary or general anaesthesia for regular and emergency caesarean section**	**0.89**	3.40 ±0.83	3.51 ±1.07	**0.91**	3.52 ±0.65	3.48 ±0.83	**0.85**	3.35 ±0.85	3.60 ±1.16	**0.89**	3.39 ±0.83	3.83 ±1.01	**0.89**	3.32 ±0.98	3.16 ±1.18

Attachment 4 to: Moll-Khosrawi P, Ganzhorn A, Zöllner C, Schulte-Uentrop L. *Development and validation of a postgraduate anaesthesiology core curriculum based on Entrustable Professional Activities: a Delphi study.* GMS J Med Educ. 2020;37(5):Doc52. DOI: 10.3205/zma001345

**Providing perioperative care for patients with ASA ≤ III**	**0.88**	3.66 ±0.69	1.50 ±0.74	**0.87**	3.65 ±0.70	1.57 ±0.77	**0.85**	3.60 ±0.73	1.55 ±0.67	**0.83**	3.61 ±0.76	1.44 ±0.83	**0.95**	3.79 ±0.52	1.42 ±0.67
**Providing anaesthetic care for small laparoscopic surgery**	**0.88**	3.54 ±0.74	1.66 ±0.77	**0.91**	3.70 ±0.62	1.78 ±0.83	**0.95**	3.70 ±0.56	1.70 ±0.84	**0.83**	3.44 ±0.76	1.78 ±0.71	**0.79**	3.26 ±0.91	1.37 ±0.58
**Providing postoperative care in the recovery room**	**0.85**	3.35 ±0.85	2.09 ±0.94	**1.00**	3.70 ±0.46	2.17 ±0.92	**0.80**	3.35 ±0.79	1.85 ±0.73	**0.94**	3.44 ±0.60	2.28 ±0.99	**0.63**	2.84 ±1.18	2.05 ±1.05
**Providing anaesthetic care for large laparoscopic surgery**	**0.85**	3.20 ±0.75	2.58 ±1.09	**0.91**	3.30 ±0.62	2.78 ±0.72	**0.80**	3.05 ±0.80	2.65 ±1.01	**0.78**	3.22 ±0.79	2.89 ±1.24	**0.89**	3.21 ±0.77	1.95 ±1.15
**Providing anaesthetic care for thoracic surgery (including lung seperation) with normal lung function**	**0.81**	3.09 ±0.74	3.30 ±0.91	**0.87**	3.13 ±0.74	3.48 ±0.65	**0.75**	2.95 ±0.67	3.25 ±1.18	**0.83**	3.22 ±0.85	3.33 ±0.88	**0.79**	3.05 ±0.69	3.11 ±0.85
**Indication, consideration and performance of spinal and epidural anaesthesia**	**0.80**	3.23 ±0.79	2.43 ±0.88	**0.78**	3.22 ±0.88	2.65 ±0.76	**0.80**	3.25 ±0.77	2.30 ±0.90	**0.78**	3.11 ±0.74	2.28 ±1.04	**0.84**	3.32 ±0.73	2.42 ±0.75
**Administer general anaesthesia in pediatric patients over the age of five**	**0.80**	3.16 ±0.87	3.60 ±1.07	**0.83**	3.17 ±0.82	3.48 ±1.06	**0.85**	3.35 ±0.73	3.50 ±0.81	**0.78**	3.17 ±0.76	3.72 ±1.19	**0.74**	2.95 ±1.10	3.74 ±1.16
**Providing anaesthetic care for intracranial surgery without increased intracranial pressure**	**0.80**	3.10 ±0.86	2.60 ±1.01	**0.87**	3.17 ±0.87	2.78 ±0.66	**0.70**	2.90 ±0.94	2.65 ±1.06	**0.78**	3.28 ±0.80	2.61 ±1.30	**0.84**	3.05 ±0.76	2.32 ±0.92
**Performing in-house transfers of critically ill patients**	**0.79**	3.23 ±0.84	2.63 ±0.95	**1.00**	3.61 ±0.49	2.78 ±0.83	**0.70**	3.15 ±0.85	2.55 ±0.67	**0.72**	3.11 ±0.81	3.06 ±1.03	**0.68**	2.95 ±1.00	2.11 ±1.02
**Management of in-house emergencies**	**0.79**	3.10 ±0.93	3.93 ±1.02	**0.70**	2.83 ±1.01	4.26 ±0.90	**0.85**	3.15 ±0.79	4.00 ±0.89	**0.78**	3.17 ±0.76	3.72 ±1.10	**0.84**	3.32 ±1.03	3.63 ±1.09
**Providing anaesthetic care for patients with severe pre-existing cardiac conditions**	**0.78**	3.15 ±0.98	3.84 ±1.06	**0.61**	2.78 ±1.21	4.09 ±0.78	**0.85**	3.20 ±0.81	3.74 ±1.02	**0.89**	3.44 ±0.68	4.06 ±0.97	**0.79**	3.26 ±0.91	3.42 ±1.31
**Providing anaesthetic care and emergency management for critically injured and ill patients in the shock room**	**0.78**	3.00 ±0.95	4.34 ±1.00	**0.65**	2.78 ±1.10	4.61 ±0.82	**0.80**	3.00 ±0.95	4.35 ±0.91	**0.89**	3.11 ±0.74	4.39 ±1.16	**0.79**	3.16 ±0.87	3.95 ±1.00

Attachment 4 to: Moll-Khosrawi P, Ganzhorn A, Zöllner C, Schulte-Uentrop L. *Development and validation of a postgraduate anaesthesiology core curriculum based on Entrustable Professional Activities: a Delphi study.* GMS J Med Educ. 2020;37(5):Doc52. DOI: 10.3205/zma001345

**Providing postoperative pain management**	**0.76**	3.13 ±0.86	2.20 ±1.16	**0.83**	3.26 ±0.74	2.26 ±0.79	**0.70**	3.10 ±0.83	2.55 ±1.63	**0.89**	3.28 ±0.65	2.06 ±0.97	**0.63**	2.84 ±1.09	1.89 ±0.97
**Providing perioperative anaesthetic and emergency care for critically injured and ill patients**	**0.76**	3.04 ±0.86	3.86 ±0.95	**0.78**	3.13 ±0.99	4.00 ±0.72	**0.80**	3.05 ±0.80	3.75 ±0.89	**0.78**	3.11 ±0.74	3.94 ±1.13	**0.68**	2.84 ±0.81	3.74 ±1.02
**Performing a premedication round (preoperative evaluation) including patient education**	**0.75**	3.29 ±0.95	1.68 ±1.00	**0.87**	3.57 ±0.82	1.70 ±0.86	**0.75**	3.30 ±0.95	1.65 ±1.06	**0.78**	3.39 ±0.83	1.67 ±0.75	**0.58**	2.84 ±1.04	1.68 ±1.26
**Providing anaesthetic management for pregnant patients**	**0.75**	3.06 ±0.84	3.24 ±1.02	**0.74**	3.09 ±0.88	3.22 ±1.06	**0.85**	3.10 ±0.77	3.25 ±0.94	**0.72**	3.11 ±0.81	3.61 ±0.95	**0.68**	2.95 ±0.89	2.89 ±0.97
**Providing anaesthetic and haemodynamic management of uterine atony**	**0.74**	3.00 ±1.07	4.61 ±1.17	**0.74**	3.09 ±0.97	4.57 ±1.10	**0.65**	2.80 ±1.17	4.75 ±1.13	**0.78**	3.00 ±1.00	5.11 ±0.99	**0.79**	3.11 ±1.12	4.05 ±1.19
**Indication and performance of analgosedation**	**0.73**	3.06 ±0.94	2.16 ±0.89	**0.83**	3.09 ±1.02	2.65 ±0.87	**0.75**	3.15 ±0.79	2.30 ±0.78	**0.67**	3.11 ±0.99	2.17 ±0.76	**0.63**	2.89 ±0.91	1.42 ±0.59
**Providing anaesthetic care for intracranial surgery with (the risk of) increased intracranial pressure**	**0.71**	2.98 ±0.89	3.66 ±1.06	**0.74**	2.96 ±1.00	3.91 ±0.93	**0.70**	2.90 ±0.83	3.75 ±0.94	**0.72**	3.17 ±0.96	3.61 ±1.21	**0.68**	2.89 ±0.72	3.32 ±1.08
**Providing perioperative care for patients with major blood loss and pre-existing coagulation disorder**	**0.71**	2.90 ±1.07	4.39 ±1.20	**0.65**	2.61 ±1.28	4.43 ±1.01	**0.70**	2.90 ±0.94	4.50 ±1.12	**0.78**	3.06 ±0.97	4.76 ±1.11	**0.74**	3.11 ±0.91	3.89 ±1.37
**Communication with relatives of critically ill patients and consultation about treatment plans**	**0.70**	2.95 ±1.12	2.45 ±1.28	**0.78**	3.17 ±0.76	2.91 ±1.38	**0.65**	2.90 ±1.30	1.90 ±1.09	**0.78**	3.06 ±0.85	2.72 ±1.19	**0.58**	2.63 ±1.38	2.21 ±1.15
**Providing perioperative care for critically injured patients with increased intracranial pressure**	**0.69**	2.78 ±1.11	4.41 ±1.09	**0.61**	2.61 ±1.24	4.52 ±0.97	**0.70**	2.80 ±1.21	4.35 ±0.85	**0.67**	2.83 ±0.83	4.56 ±1.12	**0.79**	2.89 ±1.02	4.21 ±1.36
**Indication and performance of a regional anaesthesia technique**	**0.68**	2.91 ±0.87	2.89 ±1.11	**0.61**	2.70 ±0.86	3.04 ±1.04	**0.65**	2.85 ±0.73	2.95 ±1.36	**0.78**	3.22 ±0.79	2.76 ±1.00	**0.68**	2.95 ±1.00	2.74 ±0.96

Attachment 4 to: Moll-Khosrawi P, Ganzhorn A, Zöllner C, Schulte-Uentrop L. *Development and validation of a postgraduate anaesthesiology core curriculum based on Entrustable Professional Activities: a Delphi study.* GMS J Med Educ. 2020;37(5):Doc52. DOI: 10.3205/zma001345

**Administer general anaesthesia in pediatric patients under the age of five**	**0.64**	2.81 ±1.05	4.43 ±0.98	**0.61**	2.74 ±1.19	4.35 ±1.00	**0.70**	2.80 ±0.75	4.45 ±0.80	**0.61**	2.94 ±0.97	4.39 ±1.01	**0.63**	2.79 ±1.20	4.53 ±1.09
**Providing anaesthetic care for thoracic surgery (including lung seperation) with limited lung function**	**0.63**	2.76 ±0.94	4.27 ±1.09	**0.57**	2.70 ±1.04	4.52 ±0.88	**0.55**	2.55 ±0.80	4.26 ±1.21	**0.72**	2.94 ±0.97	4.17 ±1.17	**0.68**	2.89 ±0.85	4.06 ±1.08
**Providing perioperative coagulation management including interpretation and therapeutical consequences of thrombelastometry**	**0.61**	2.61 ±1.03	3.88 ±1.26	**0.61**	2.52 ±1.02	3.87 ±1.15	**0.45**	2.45 ±1.07	4.40 ±1.20	**0.67**	2.78 ±0.92	4.06 ±0.97	**0.74**	2.74 ±1.07	3.16 ±1.35
**Providing anaesthetic care for pregnant patients with HELLP/pre-eclampsia/eclampsia**	**0.58**	2.66 ±1.06	4.40 ±1.21	**0.61**	2.78 ±1.10	4.35 ±0.96	**0.45**	2.40 ±1.02	4.50 ±1.07	**0.67**	2.78 ±1.08	4.94 ±1.08	**0.58**	2.68 ±0.98	3.84 ±1.46
**Administer general anaesthesia in neonatal patients**	**0.45**	2.25 ±1.32	5.28 ±0.94	**0.39**	2.09 ±1.38	5.13 ±1.12	**0.30**	1.85 ±1.01	5.55 ±0.67	**0.56**	2.50 ±1.42	5.28 ±0.73	**0.58**	2.63 ±1.27	5.16 ±1.04
**Indication and performance of ultrasound use and diagnostic (a.e FAST) and therapeutic consequences**	**0.44**	2.34 ±1.06	3.63 ±1.26	**0.43**	2.26 ±1.11	3.91 ±1.28	**0.25**	2.15 ±0.85	3.75 ±1.13	**0.50**	2.44 ±0.76	3.67 ±1.15	**0.58**	2.53 ±1.35	3.11 ±1.29
**Providing perioperative care for patients undergoing cardiothoracic surgery**	**0.28**	1.96 ±1.16	4.70 ±0.89	**0.13**	1.57 ±1.14	4.83 ±0.87	**0.15**	1.60 ±0.97	4.50 ±0.92	**0.39**	2.33 ±1.11	4.94 ±0.70	**0.47**	2.47 ±1.09	4.53 ±0.94

Table note: The EPAs are listed downwards regarding their CVI: *"High scoring"(CVI >0.75) and "low scoring" (CVI <0.75).* ***Abbreviations:*** *YR: Year in which EPA should be conducted by the trainee without direct supervision. SD: Standard deviation. MV: Mean value. CVI: content validity index*

Attachment 4 to: Moll-Khosrawi P, Ganzhorn A, Zöllner C, Schulte-Uentrop L. *Development and validation of a postgraduate anaesthesiology core curriculum based on Entrustable Professional Activities: a Delphi study.* GMS J Med Educ. 2020;37(5):Doc52. DOI: 10.3205/zma001345
