# Supplementary material for: Development and validation of a postgraduate anaesthesiology core curriculum based on Entrustable Professional Activities: a Delphi study
Source: GMS J Med Educ. 2020 Sep 15;37(5):Doc52. doi: 10.3205/zma001345 (PMC7499458; doi:10.3205/zma001345)
Supplement: Comparison of our 39 EPA list with the EPA list of Wisman-Zwarter et al [file JME-37-52-s-005.pdf]

<u>Attachment 5</u>: Comparison of our 39 EPA list with the EPA list of Wisman-Zwarter et al.

Our EPA list is not transferable one-by-one to the list of Wisman-Zwarter et al. Nevertheless, we detected a 73.3 %. agreement with our list of EPAs. 26.6% of the list of Wisman-Zwarter et al. is not included in our EPAs due to the fact that we have excluded EPAs concerning intensive care medicine and advanced pain management.
A detailed comparison of the both EPA lists is provided in the *table below*.

Comparison of EPAs from Wisman-Zwarter et al. and our EPAs

**EPAs of Wisman-Zwarter et al.**	**EPAs of Moll-Khosrawi et al.**
Cardiopulmonary resuscitation of the adult patient	Management of in-house emergencies
Cardiopulmonary resuscitation of the paediatric patient	Management of in-house emergencies
Epidural and spinal anaesthesia	Indication, consideration and performance of spinal and epidural anaesthesia
Interviewing the patient	Performing a premedication round (preoperative evaluation) including patient education
Management of acute pain	Providing postoperative pain management
Management of chronic pain	Providing postoperative pain management
Management of massive blood loss	Haemodynamic management of major blood loss
Management of oncological pain and palliative care	Not included specifically *
Management of the difficult airway	Providing general anaesthesia including airway management in patients with anticipated airway

	Management of the unanticipated difficult airway
Obtain central venous access	Specified as OPA
Perioperative anaesthetic care for abdominal vascular surgery	Providing anaesthetic care for extensive open abdominal surgery
Perioperative anaesthetic care for ASA I-II patients undergoing high risk surgery<br><br>Perioperative anaesthetic care for ASA III patients undergoing high risk surgery<br><br>Perioperative anaesthetic care for ASA I-II patients undergoing low to medium risk surgery<br>Perioperative anaesthetic care for ASA III patients undergoing low to medium risk surgery<br>Perioperative anaesthetic care for ASA IV patients undergoing low to medium risk surgery<br>Perioperative anaesthetic care for ASA IV-V patients undergoing high risk surgery	*Subsummed in following EPAs:*<br>Providing perioperative care for patients ASA I-III<br>Providing perioperative care for patients ASA > III
Perioperative anaesthetic care for caesarean section	Indication of anaesthetic technique and performance of medullary or general anaesthesia for regular and emergency caesarean section
Perioperative anaesthetic care for children between one and four years old	Administer general anaesthesia in pediatric patients under the age of five
Perioperative anaesthetic care for children over four years of age	Administer general anaesthesia in pediatric patients over the age of five

Perioperative anaesthetic care for coronary artery bypass grafting and valve surgery	Providing perioperative care for patients undergoing cardiothoracic surgery
Perioperative anaesthetic care for day care surgery	Not included specifically*
Perioperative anaesthetic care for head and neck surgery, excluding neurosurgery and vascular surgery	Not included specifically*
Perioperative anaesthetic care for infants up to the age of one year	Administer general anaesthesia in neonatal patients
Perioperative anaesthetic care for laparoscopic surgery in day care	Providing anaesthetic care for small laparoscopic surgery<br>Providing anaesthetic care for large laparoscopic surgery<br>*(day care is not included specifically)*
Perioperative anaesthetic care for laryngotracheobronchoscopy in adults and children	Not included specifically*
Perioperative anaesthetic care for non-vascular abdominal surgery	Not included specifically*
Perioperative anaesthetic care for peripheral vascular surgery	Not included specifically*
Perioperative anaesthetic care for prosthetic and osteosynthetic surgery of the knee or hip	Not included specifically*
Perioperative anaesthetic care for pulmonary surgery	Providing anaesthetic care for thoracic surgery (including lung seperation) with normal lung function<br>Providing anaesthetic care for thoracic surgery (including lung seperation) with limited lung function

Perioperative anaesthetic care for situations of a shared airway with the surgical team	Not included specifically*
Perioperative anaesthetic care for vascular surgery of the Carotid artery	Not included specifically*
Peripartum pain management	Indication of anaesthetic technique and performance of medullary or general anaesthesia for regular and emergency caesarean section<br>(Not specifically, former EPA "*Providing epidural anaesthesia during labour*", was merged into the above mentioned)
Peripheral nerve block	Indication and performance of a regional anaesthesia technique
Post-acute and long-term intensive care	Not included specifically*
Postoperative care during the recovery period	Providing postoperative care in the recovery room
Postoperative intensive care after (cardiac) surgery in the post-anaesthesia care unit (PACU)	Providing postoperative care in the recovery room
Preoperative assessment	Performing a premedication round (preoperative evaluation) including patient education
Resuscitation and admission of the adult patient in need of intensive care	Performing in-house transfers of critically ill patients<br>Management of in-house emergencies<br>Providing perioperative anaesthetic and emergency care for critically injured and ill patients

Resuscitation and treatment of sepsis in the intensive care unit	Management of in-house emergencies
Resuscitation of the acutely ill paediatric patient	Management of in-house emergencies
Resuscitation of the adult multiple trauma patient in the Emergency Room	Providing anaesthetic care and emergency management for critically injured and ill patients in the shock room<br>Management of in-house emergencies
Science and evidence based medicine	Not included specifically*
Sedation for medical interventions and examinations	Indication and performance of analgosedation
Ventilation on the intensive care unit	Not included specifically*

**These EPAs were not mentioned specifically in our list of EPAs, therefore they were not included into the calculation of agreement.*
